# Photoswitchable Activity of Mixed Bismuth Oxide (BiO_*x*_) for Water Splitting in Neutral Media

**DOI:** 10.1021/acsomega.5c10847

**Published:** 2026-03-04

**Authors:** André Guimarães de Oliveira, Aparecida Cristina Mauro, Marcus Vinicius David, Ana Maria Rocco

**Affiliations:** 1 Conductive Materials and Energy Group, Chemical and Biochemical Engineering Processes, School of Chemistry, 341391 UFRJ, Universidade Federal do Rio de Janeiro, Centro de Tecnologia, Bloco E,Rio de Janeiro, RJ 21941-909, Brazil; 2 Materials Metrology Division, National Institute of Metrology, Standardization and Industrial Quality, Inmetro/Dimat, Av. N. S. Graças 50, Xerém, Sao Paolo, RJ 25250-020, Brazil

## Abstract

Unary bismuth oxide (BiO_*x*_), containing both Bi^3+^ and Bi^5+^ species, exhibits a photoswitchable response that, to the best of our knowledge, has not yet been reported. The material has attracted increasing attention as an anode for the oxygen evolution reaction (OER), often in combination with additional elements to enhance electrochemical performance. This study investigates the electrodeposition of BiO_*x*_ and its unexplored photoswitchable properties as an anode for solar-driven enhanced water splitting. Illumination with a xenon lamp equipped with solar simulation filters reduced the deposition time from 90 to 10 min without altering the amperometric profile, offering significant energy savings and potential for scale-up. Structural and morphological characterization were performed using scanning electron microscopy with energy-dispersive X-ray spectroscopy (SEM-EDS) and X-ray diffraction (XRD). Chopped light voltammetry revealed that the photocurrent in neutral media has a mixed photo- and electrocatalytic contribution. Under illumination, the BiO_*x*_ electrode achieved a total current density of 2.5 mA cm^–2^, 140 mV lower than in dark conditions. Electrochemical impedance spectroscopy (EIS) revealed a decrease in depletion layer resistance with illumination, and an electrolyte-free impedance measurement indicated that changes in intrinsic conductivity also contribute to the PEC response. Overall, illumination enhances BiO_*x*_ electrocatalytic water splitting, with a particularly pronounced effect on thin-layer films.

## Graphical abstract

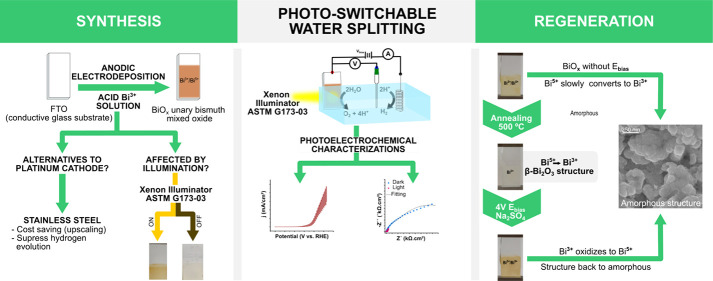

## 1 Introduction

Sustainable green hydrogen production requires large-scale capacity and competitive cost to become a viable pathway in the global energy transition. A major challenge in developing efficient systems lies in the oxygen evolution reaction (OER), which is intrinsically sluggish due to its complex multielectron transfer steps.^1–6^ Addressing this kinetic bottleneck is essential for advancing water-splitting technologies. Metal oxides (MO) are considered “classic” anode materials and have been extensively studied for the OER owing to their demonstrated scalability, robust performance, and well-established synthesis routes.^7–10^ These materials offer several advantages, including chemical stability, earth abundance, and tunable electronic properties, making them promising candidates for long-term water oxidation. MOs are suitable for both electrochemical^11,12^ and photoelectrochemical (PEC)^13–18^ water splitting.

Bismuth-based oxides have recently attracted considerable attention due to their favorable band edge positions, visible-light absorption, and relatively low environmental impact.^19,20^ Among them, bismuth vanadate (BiVO_4_) stands out as the most extensively investigated bismuth-based photoanode owing to its narrow bandgap (∼2.4 eV), strong optical absorption, and chemical stability.^21–24^ Despite these advantages, BiVO_4_ suffers from limited charge transport and surface recombination issues, often requiring the use of cocatalysts or doping strategies to enhance its performance.^7,25,26^ Beyond BiVO_4_, other bismuth-containing compounds—such as bismuth oxyhalides (BiOX, where X = Cl, Br, I) and bismuth ferrite (BiFeO_3_)—have demonstrated favorable electronic properties.^27,28^ The presence of photoswitchable oxygen vacancies capable of generating catalytically active sites has also been reported for these materials, an effect that has been explored in photocatalysis,^29,30^ but remains unexamined in electrocatalytic systems. Recent studies have demonstrated that unary bismuth oxide (BiO_*x*_) can exhibit intrinsic OER activity through the reversible Bi^3+^/Bi^5+^ redox couple,^31^ as other noninnocent metal species present in metal oxides,^32^ which enables catalytic activity in acidic media over extended periods.^33–35^

The fabrication method also plays a critical role in electrode development; a scalable processing is required for large-scale green hydrogen production. Although techniques such as sputtering and atomic layer deposition (ALD) provide materials with a higher degree of organization, characterized by low intergranular and lattice defects, their large-scale implementation faces technical and economic challenges.^36^ Alternatively, scalable deposition strategies, such as dip-coating, spin-coating, spray-coating, doctor blading, slot die casting, screen printing, inkjet printing, and aerosol jet printing, have been employed for the fabrication of metal oxide-based electrodes.^37^ Solution-based methods, including hydrothermal synthesis and electrodeposition, offer cost-effective and scalable routes.^38^ Electrodeposition is advantageous due to its simplicity, low material consumption, and improved film–substrate adhesion.^39,40^ However, to render the process suitable for scale-up, it is essential to minimize complexity and avoid the use of costly materials.

In this work, we aimed to develop a cost-effective and scalable method for BiO_*x*_ electrodeposition by using a single-compartment electrochemical cell with a stainless steel counter electrode in acidic media to enhance oxide growth. We also investigated the effect of illumination during acid electrodeposition. Further, the photoswitchable performance of BiO_*x*_ in neutral electrolyte was evaluated using chopped light voltammetry and electrochemical impedance spectroscopy (EIS). To assess the contribution of intrinsic conductivity changes to the photocurrent, an electrolyte-free impedance experiment was designed to compare BiO_*x*_ and BiVO_4_. To the best of our knowledge, this is the first report in the literature exploring the photoswitchable activity of BiO_*x*_ as an anode for water splitting.

## 2 Results and Discussion

### 2.1 BiO_*x*_ Electrodeposition

Figure 1 shows the plot of current density as a function of time for electrodeposition of BiO_*x*_ on FTO (WE, anode) from a solution containing 0.2 M BiNO_3_·5H_2_O in HNO_3_ 0.63 and 7 M acetic acid, applying a potential of 2.57 V (vs Hg_2_Cl_2_). The electrodeposition of metal oxides is commonly performed under neutral or mildly acidic conditions, which minimizes the potential required for oxide formation, as indicated by Pourbaix diagrams.^41^ However, lower pH solutions may cause the dissolution of metal oxides, which impacts material stability for long-term applications.^42^ When applied to the oxygen evolution reaction (OER), neutral or alkaline solutions also pose challenges, such as carbon dioxide uptake and the precipitation of carbonates, issues that are absent in acidic media. Electrodeposition of metal oxides in an acidic medium is advantageous, producing stable materials for OER in low pH solutions. Wang *et al*.^43^ reported the electrodeposition of bismuth oxide in a highly acidic solution (pH = 0.44). However, their study lacked a detailed mechanistic investigation, which motivated the present work to gain a better understanding of this process.

**Figure 1:**
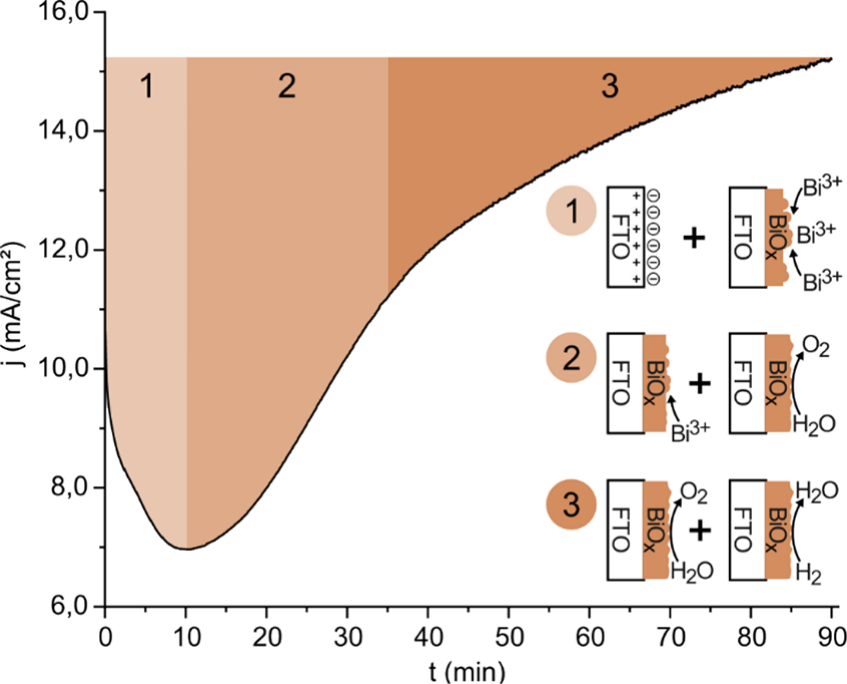
Chronoamperometric curve obtained during the electrodeposition of BiO_*x*_ (no illumination). *E*_applied_ = 2.57 V vs Hg_2_Cl_2_. Three-electrode cell: WE, FTO; RE, Hg_2_Cl_2_ (3 M); and CE, stainless steel. Electrodeposition solution 0.2 M BiNO_3_·5H_2_O in HNO_3_ 0.63 and 7 M acetic acid. Regions 1, 2, and 3 illustrate the electrochemical processes occurring on the WE surface.

Electrodeposition recorded under constant potential exhibits characteristics consistent with the formation of transition metal oxides (Figure 1), as described by Di Girolamo *et al*. for NiOOH.^44^ Given the high positive potential applied to the working electrode, multiple oxidation reactions can occur at the electrode surface depending on the electrolyte composition, as shown below. It is important to note that, under the experimental conditions, Bi^4+^ ions are not thermodynamically stable, and eq 4 is therefore considered the general reaction describing Bi^5+^ formation.

$$2H_{2}O_{\left(l\right)}\to O_{2(g)}+4H^{+}+4e^{-}$$ 1

$${\mathrm{Bi}}_{\left(\mathrm{aq}\right)}^{3+}\to {\mathrm{Bi}}^{4+}+e^{-}$$ 2

$${\mathrm{Bi}}^{4+}\to {\mathrm{Bi}}^{5+}+e^{-}$$ 3

$${\mathrm{Bi}}_{\left(\mathrm{aq}\right)}^{3+}\to {\mathrm{Bi}}^{5+}+2e^{-}$$ 4

$${\mathrm{CH}}_{3}{\mathrm{COOH}}_{\left(\mathrm{aq}\right)}+2H_{2}O_{\left(l\right)}\to 2{\mathrm{CO}}_{2(g)}+8H^{+}+8e^{-}$$ 5

Three different regions might be observed during electrodeposition. In the first region (0–10 min), a current drop is observed due to double-layer charging and the depletion of reactants near the electrode surface. Wang *et al*.^43^ mention the possible oxidation of acetic acid under experimental conditions (high potential and significant acid concentration). Notably, the low pH prevents the formation of HCO_3_^–^ and CO_3_^2–^, making CO_2_ the main reaction product, as described in eq 5.

As the reactant concentration decreases, the second region (10–35 min) is dominated by the nucleation-driven growth of BiO_*x*_ (eqs 2–4). BiO_*x*_ exhibits catalytic activity toward the OER (eq 1). This activity, combined with the amorphous nature of the growing oxide, which enhances the electrode’s active surface area, leads to a pronounced increase in the measured current. At this point, the film becomes easily visible to the naked eye.

In the third region (35–90 min), the slope of the curve decreases as most of the bismuth is consumed at both the anode and cathode. Bubble formation becomes evident at the counter electrode. The increasing current during this phase can be attributed to the oxidation of hydrogen diffusing from the counter electrode, co-occurring with the OER. By the end of the experiment, a brown BiO_*x*_ film is visibly deposited on the FTO surface (Figure 5).

At the counter electrode, the reduction of Bi^3+^ to Bi_(s)_ is evidenced by the formation of a thick, dark film, with minimal hydrogen bubbles observed initially. Compared to Pt, a stainless-steel counter electrode offers advantages, including lower cost and reduced catalytic activity for hydrogen generation. Using a single-cell setup for electrodeposition, molecular hydrogen diffuses from the counter electrode to the working electrode, where it can be oxidized, competing with oxide deposition. Experiments employing Pt as the counter electrode in the same conditions resulted in a less visible oxide layer (not shown). In contrast, stainless steel produced a visibly thicker oxide film, suggesting that higher molecular hydrogen concentration in the electrolyte might inhibit BiO_*x*_ growth.

The following reactions summarize the processes occurring at the counter electrode. It is important to note that, under the experimental conditions, Bi^2+^ and Bi^+^ ions are not thermodynamically stable, and eq 9 is therefore considered the general reaction describing the formation of metallic Bi_(s)_.

$${\mathrm{Bi}}_{\left(\mathrm{aq}\right)}^{3+}+e^{-}\to {\mathrm{Bi}}^{2+}$$ 6

$${\mathrm{Bi}}^{2+}+e^{-}\to {\mathrm{Bi}}^{+}$$ 7

$${\mathrm{Bi}}^{+}+e^{-}\to {\mathrm{Bi}}_{\left(s\right)}$$ 8

$${\mathrm{Bi}}_{\left(\mathrm{aq}\right)}^{3+}+3e^{-}\to {\mathrm{Bi}}_{\left(s\right)}$$ 9

$$2H_{\left(\mathrm{aq}\right)}^{+}+2e^{-}\to H_{2(g)}$$ 10

The catalytic effect observed during BiO_*x*_ electrodeposition in the OER can be attributed to the noninnocent role of bismuth oxide, as discussed by Thorarinsdottir *et al*.^31^ These authors emphasized the critical role of the Bi^3+^/Bi^5+^ redox couple in the OER mechanism. Our experiments further revealed a pronounced photoresponse for this material, indicating a photoswitchable activity that could be related either to changes in the material’s conductivity or to the presence of photoswitchable oxygen vacancies.

The film’s photoresponse is evident even during electrodeposition. Chopped illumination experiments (Figure 2) exhibited a nearly 4-fold increase in current between dark and illuminated conditions with 19.5 min of electrodeposition. The periodic light pulses directly compared dark and illuminated conditions within the same experiment. While the dark current (red) followed the same profile as the electrodeposition without illumination (Figure 1), the light current (blue) exhibited a distinct profile with significantly higher currents (80% of the maximum current was reached at 17.1 min of electrodeposition).

**Figure 2:**
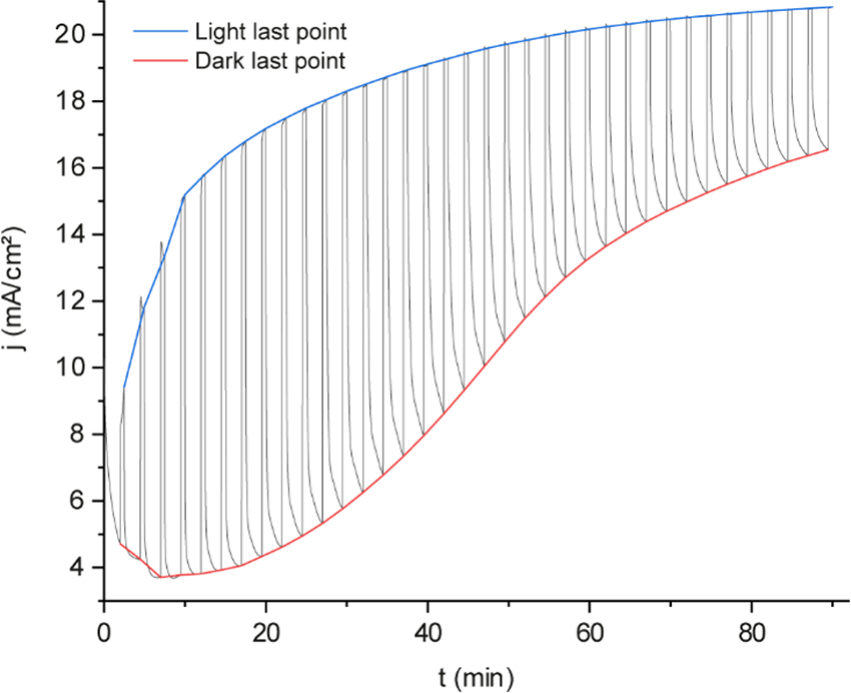
Chronoamperometric curve obtained during the electrodeposition of BiO_*x*_ (chopped illumination). *E*_applied_ = 2.57 V vs Hg_2_Cl_2_. *t*_deposition_ = 90 min. Three-electrode cell: WE, FTO (back illumination); RE, Hg_2_Cl_2_ (3 M); and CE, stainless steel. Electrodeposition solution 0.2 M BiNO_3_·5H_2_O in HNO_3_ 0.63 and 7 M acetic acid. The blue curve connects the last point of each current measurement with the light source shutter off. The red curve connects the last point of each current measured with the light source shutter on.

Over time, the difference between dark and illuminated conditions diminishes as the dark current increases due to the electrocatalytic response of BiO_*x*_, while the photocurrent does not follow a similar trend. An increase in electron–hole recombination may explain this behavior as the BiO_*x*_ layer thickness grows. For each light pulse in Figure 2, the current difference (Δ*j*) between dark and illuminated conditions was calculated, revealing a maximum Δ*j* at 22.2 min (Figure 3). This result indicates that it is possible to reduce deposition time with external illumination.

**Figure 3:**
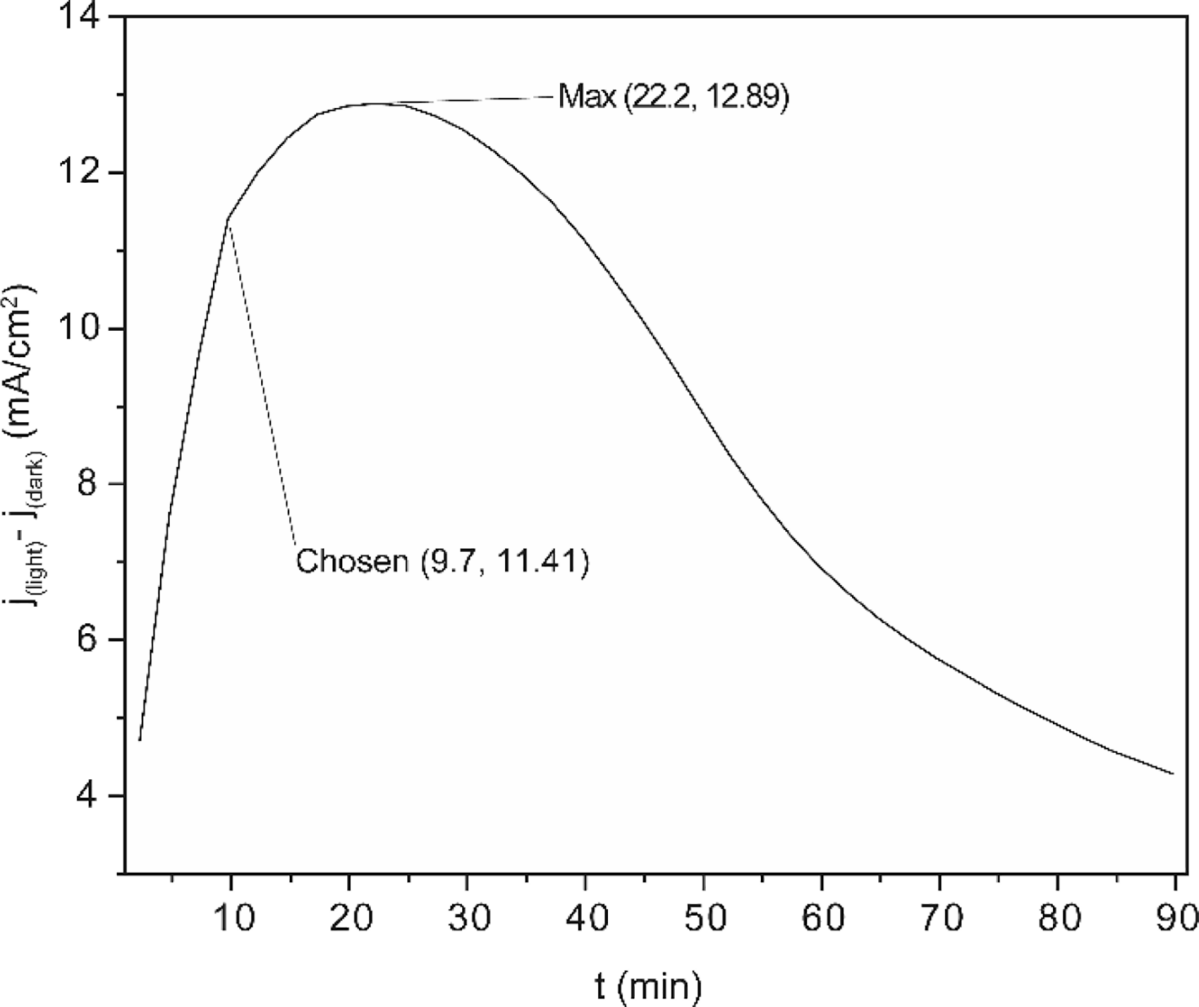
Delta current (*j*_(light)_ – *j*_(dark)_) versus electrodeposition time. Data was obtained by subtracting the blue and red curve of Figure 2.

Figure 4 shows the 10 min electrodeposition curves with (blue) and without (red) constant illumination. The result obtained with illumination contains all electrodeposition steps observed in the 90 min electrodeposition without illumination (Figure 1). On the other hand, 10 min electrodeposition without illumination only exhibited the previously observed current drop, indicating minimal material growth. The results confirm that external illumination might reduce electrodeposition time.

**Figure 4:**
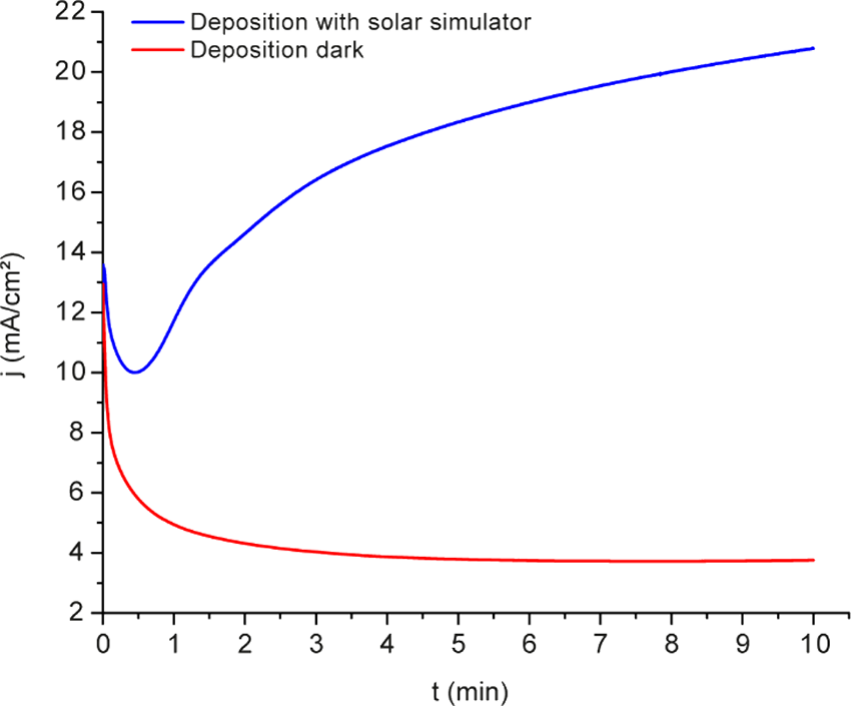
Chronoamperometric curves obtained during the electrodeposition of BiO_*x*_ with (blue curve) and without (red curve) illumination. *E*_applied_ = 2.57 V vs Hg_2_Cl_2_. *t*_deposition_ = 10 min. Three-electrode cells: WE, FTO; RE, Hg_2_Cl_2_ (3 M); and CE, stainless steel. Electrodeposition solution 0.2 M BiNO_3_·5H_2_O in HNO_3_ 0.63 and 7 M acetic acid.

The photoelectrodes obtained after electrodeposition exhibited distinct visual characteristics, as shown in Figure 5. Electrodes deposited for 90 min appeared dark brown, whereas those deposited for 10 min exhibited a lighter brown coloration. A noticeable difference was observed in the photoelectrode deposited for 10 min without illumination, which displayed a very light brown deposit compared to the electrode deposited under the same conditions but under illumination. This difference suggests a lower amount of deposited BiO_*x*_, a hypothesis further supported by subsequent characterizations. Due to the intrinsic instability of Bi^5+^ species within the oxide, the BiO_*x*_ film gradually becomes lighter in color upon exposure to air as it undergoes partial conversion to Bi_2_O_3_. Annealing the film at 500 °C for 2 h accelerates the complete conversion into Bi_2_O_3,_ producing an opaque white color, the formation of the Bi_2_O_3_ crystalline phase is subsequently confirmed by XRD analysis (Section 2.2). The reversible nature of the Bi^3+^/Bi^5+^ redox couple allows BiO_*x*_ regeneration through anodic polarization. Thorarinsdottir *et al*.^31^ demonstrated this regeneration in a 10 mM H_2_SO_4_ electrolyte. Similar results were obtained with 1 M Na_2_SO_4_ using a two-electrode configuration. The electrodes were polarized at 4 V for 10 min to restore the as-deposited film color.

**Figure 5:**
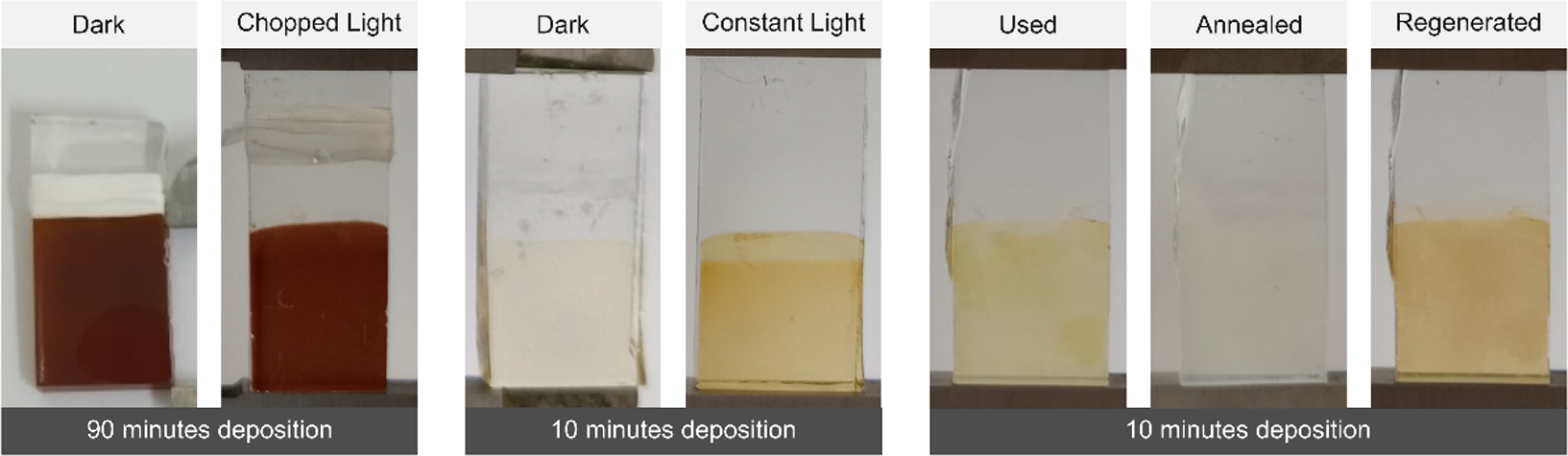
Photoelectrodes obtained after: 90 min of electrodeposition without illumination and with chopped illumination; 10 min of electrodeposition with constant illumination and no illumination; 10 min of electrodeposition after chopped light voltammetry and exposition to air (used), after annealing at 500 °C for 2 h and after regeneration at 4 V for 10 min in 1 M Na_2_SO_4_ using a two-electrode cell with a stainless steel electrode and a DC power supply.

### 2.2 BiO_*x*_ Structure and Morphology

Figure 6 shows scanning electron microscopy (SEM) images, which provide detailed insights into the surface morphology and microstructural features of the electrodeposited material. Low-amplification images exhibit dark spots where FTO is still exposed, indicating that the BiO_*x*_ did not fully cover the substrate. Nevertheless, homogeneous deposition of BiO_*x*_ is also observed in considerable areas of the substrate. With higher amplification in the electrodeposited regions, a cauliflower-shaped structure is visible for BiO_*x*_, a characteristic of materials that exhibit fractal growth. The film morphology is generally rough and amorphous,^31^ a characteristic confirmed by XRD analysis.

**Figure 6:**
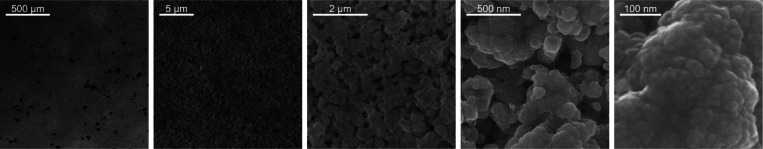
SEM images of BiO_*x*_ films deposited on FTO substrate with 130×, 10,000×, 40,000×, 160,000×, and 600,000× magnifications (left to right).

Complementary analysis using energy-dispersive X-ray spectroscopy (EDS, Figures S2–S4) confirmed the presence of key elements in the film composition and spatial distribution, verifying the presence of Bi and O in BiO_*x*_ deposition. Sn, F, and Si atoms are also present due to the FTO substrate. EDS also revealed the presence of carbon residues in the dark spots where BiO_*x*_ was not electrodeposited, indicating that residues on the substrate surface may have diminished the oxide electrodeposition.

X-ray diffraction (XRD) analysis provided structural information on electrodeposited BiO_*x*_ on the FTO substrate (Figure 7). The as-deposited BiO_*x*_ sample (pink) exhibits an amorphous nature, as evidenced by the absence of distinct diffraction peaks from the FTO substrate (black). This characteristic suggests a lack of long-range crystallinity in the as-deposited film, a common feature of electrodeposited materials due to their rapid nucleation and growth kinetics.

**Figure 7:**
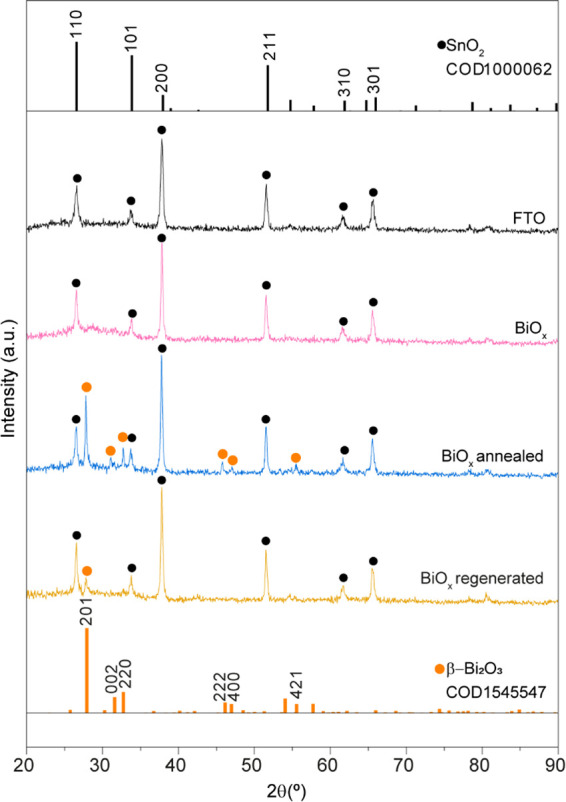
XRD patterns of FTO (black), BiO_*x*_ as deposited (pink), BiO_*x*_ after annealing (blue), and BiO_*x*_ after regeneration (dark yellow). XRD reference patterns from Crystallography Open Database (COD) of SnO_2_ (black bars) and β-Bi_2_O_3_ (orange bars).

Annealing at 500 °C improved the crystallinity of the BiO_*x*_ film. Upon annealing, a noticeable color change occurred, transitioning from brown to an opaque white. The crystallization of BiO_*x*_ resulted in the formation of β-Bi_2_O_3_, as confirmed by the emergence of multiple diffraction peaks (blue), which align with the reference pattern for β-Bi_2_O_3_ (COD 1545547). Although this improvement in crystallinity could potentially enhance the material’s electronic properties, the photo- and electrocatalytic activities of BiO_*x*_ diminished following the transition to Bi_2_O_3_. Consequently, annealing treatment was not applied prior to photoelectrochemical characterization.

BiO_*x*_ regeneration through anodic polarization reduced the film’s crystallinity, restoring the amorphous nature of the film, as Bi^5+^ species are generated within the oxide structure. Consequently, the diffractogram only exhibits the strongest β-Bi_2_O_3_ diffraction peak (201 Miller index).

### 2.3 Chopped Light Voltammetry

After verifying the photoresponse of BiO_*x*_ during electrodeposition under acidic conditions, the behavior of films electrodeposited for 10 min was further investigated in neutral media using a 1 M Na_2_SO_4_ solution. From this point forward, BiO_*x*_ electrodes will be distinguished as BiO_*x*_-D and BiO_*x*_-L, corresponding to the absence or presence of illumination during the electrodeposition process.

Chopped light voltammetry (Figure 8) was performed starting at each sample’s open circuit potential (OCP) and scanned up to 2.55 V vs RHE. Starting the measurements at the OCP avoided cathodic polarization, while the selected final potential enabled the investigation of the system until significant anodic currents were observed. Consequently, the voltammograms in Figure 8 do not initiate at the same potential relative to the RHE.

**Figure 8:**
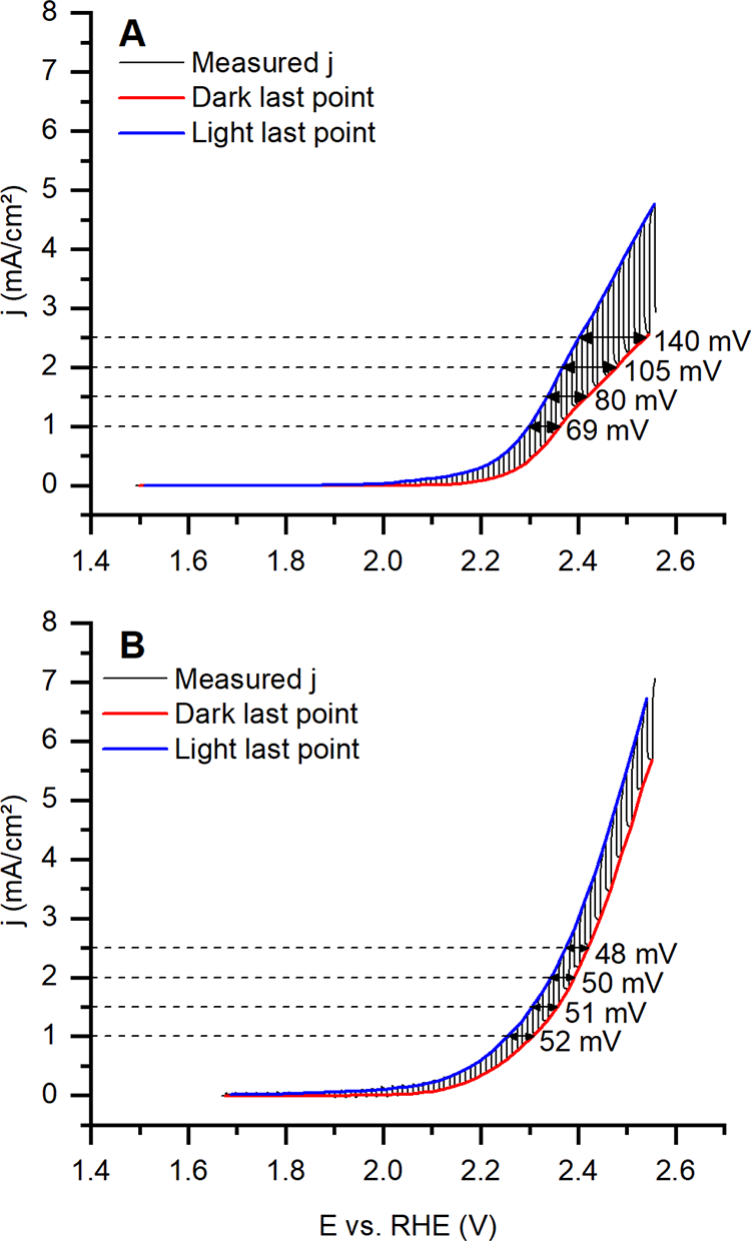
Chopped light voltammetry with three electrode cells: WE, BiO_*x*_ samples; RE, Hg_2_Cl_2_ (3 M); and CE, Pt spiral. Na_2_SO_4_ 1 M solution. Insets: (A) Working electrode BiO_*x*_-D (electrodeposited without illumination). (B) Working electrode BiO_*x*_-L (electrodeposited with illumination). The blue curve connects the last point of each current measurement with the light source shutter off. The red curve connects the last point of each current measured with the light source shutter on.

BiO_*x*_ electrodes exhibited OCP values higher than 1.5 V vs RHE in Na_2_SO_4_ solution. The high OCP can be associated with the presence of Bi^5+^ species, which have a strong tendency to be reduced. Considering that BiO_*x*_-L has a higher amount of Bi^5+^ compared to BiO_*x*_-D, this explains why it exhibits a higher OCP and a lower overpotential to achieve 1 mA/cm^2^ (Table 1).

**Table 1:** Key Values Extracted from Chopped Light Voltammetry^a^

sample	OCP (V vs RHE)	η_light_ at 1 mA/cm^2^ (V)	η_dark_ at 1 mA/cm^2^ (V)	Δ*E*_(dark‑light)_ at 2.5 mA/cm^2^ (mV)
BiO_*x*_-D	1.540	0.754	0.823	140
BiO_*x*_-L	1.716	0.540	0.592	48

a Open circuit potential (OCP); overpotential (η).

Regarding the photoresponse of each electrode, Figure 8 indicates that although BiO_*x*_-L presented a response to illumination, the potential shift from light to dark to achieve the same current density was almost constant, with a slight decrease as the electrode potential increased. The situation was different for BiO_*x*_-D, where the same potential shift increased from 69 mV at 1 mA/cm^2^ to 140 mV at 2.5 mA/cm^2^. The difference between the materials may be explained by the stronger electron–hole recombination inside BiO_*x*_-L, a consequence of the thicker layer of material deposited.

### 2.4 Electrochemical Impedance Spectroscopy

Transition metal oxides are inherently poor electrical conductors, as reflected in their high impedance values, particularly under dark conditions when both the depletion and Helmholtz layers play a significant role. Due to the semiconducting nature of the studied materials, illumination induces a reduction in impedance associated with the depletion layer, while the values associated with Helmholtz layer impedance remains significantly less affected. This effect is clearly demonstrated in Figure 10B, where the BiO_*x*_-L shows a 10-fold reduction in total impedance. Notably, the first semicircle—attributed to charge transfer processes within the depletion layer—is distinctly resolved.

Figure 9 illustrates the electrical circuit model used for EIS data fitting, which accounts for electrolyte resistance (Rs) in series with two charge transfer processes, each represented by a parallel resistor–constant phase element (CPE) pair. The first process corresponds to the depletion layer of the semiconductor (*R*_DP_ and *Q*_DP_), while the second is associated with the Helmholtz layer at the electrode/electrolyte interface (*R*_H_ and *Q*_H_). Constant phase elements were converted into effective capacitances for further analysis.

**Figure 9:**
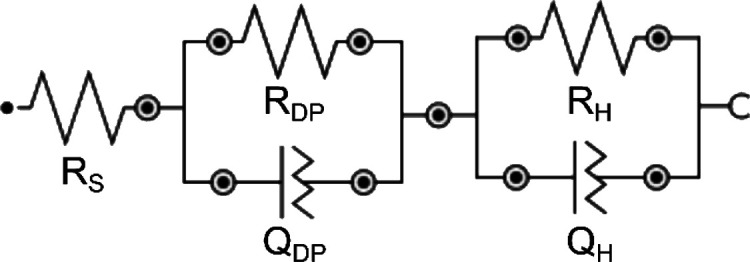
Equivalent electrical circuit model used to fit EIS data of the electrochemical cell, acquired using BiO_*x*_.

For BiO_*x*_-D (Figure 10A), a significant decrease in *R*_DP_ was observed under illumination (Table 2). However, Nyquist plots reveal substantially higher impedance values than BiO_*x*_-L, despite the lower amount of deposited oxide in BiO_*x*_-D. This discrepancy may stem from the spontaneous conversion of BiO_*x*_ into Bi_2_O_3_, a process that occurs when the electrode is not subjected to high polarization, thereby influencing the material’s behavior. All impedance measurements were conducted immediately after electrodeposition to minimize this effect. Nevertheless, the smaller quantity of material deposited in BiO_*x*_-D may have contributed to an accelerated conversion to Bi_2_O_3_.

**Figure 10:**
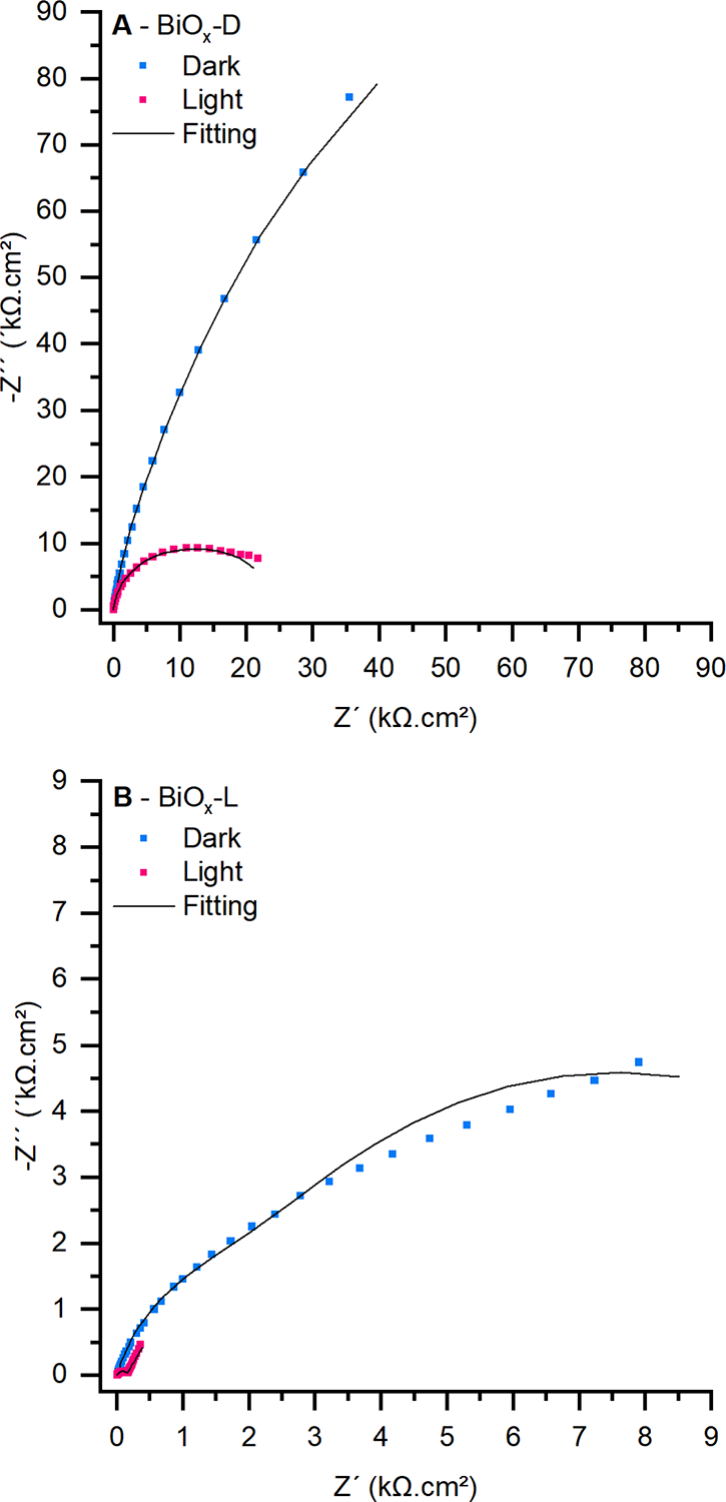
EIS spectra in dark and illuminated conditions with three electrode cells: WE, BiO_*x*_ samples; RE, Hg_2_Cl_2_ (3 M); and CE, Pt spiral. Na_2_SO_4_ 1 M solution. (A) Working electrode BiO_*x*_-D (electrodeposited without illumination). (B) Working electrode BiO_*x*_-L (electrodeposited with illumination). Parameters: *E*_DC_ = OCP; amplitude = 20 mV; frequency range = 10 kHz to 0.1 Hz. Straight line, nonlinear regression fitting using the electrical circuit analog from Figure 9.

**Table 2:** Equivalent Circuit Analog Values after Fitting Experimental EIS Results

sample	OCP (V vs RHE)^a^	*R*_s_ (Ω)	*R*_DP_ (Ω)	*C*_DP_ (F)/n^b^	*R*_H_ (Ω)	*C*_H_ (F)/n^b^
EIS with solar simulator off (dark)						
BiO_*x*_-L	1.791	12.43	1967	1.49 × 10^–5^/0.86	1.17 × 10^4^	7.50 × 10^–5^/0.83
BiO_*x*_-D	1.620	11.87	2.35E5	1.69 × 10^–5^/0.99	1.36 × 10^4^	4.08 × 10^–5^/0.85
EIS with solar simulator on (light)						
BiO_*x*_-L	1.679	12.23	132.4	9.60 × 10^–6^/0.88	1.43 × 10^4^	1.82 × 10^–3^/0.68
BiO_*x*_-D	1.502	11.92	8800	1.59 × 10^–5^/0.97	1.59 × 10^4^	3.70 × 10^–5^/0.88

a Open circuit potential (OCP).

b Effective capacitance was calculated from constant phase elements.

The results in Table 2 indicate that *C*_DP_ and *R*_H_ remained within the same order of magnitude under dark and illuminated conditions. In contrast, *R*_DP_ varied by more than an order of magnitude. BiO_*x*_-L exhibited a pronounced increase in *C*_H_, suggesting enhanced charge accumulation at the electrical double layer under illumination. These findings underscore the significant impact of photoactivation on the system’s redox dynamics, particularly as evident in the *R*_DP_ response.

### 2.5 Electrolyte Free Impedance Spectroscopy

An electrolyte-free impedance experiment was designed to study the photoresponse of the material in the absence of an electrochemical interface. This way, it can be evaluated whether the current increase under illumination originates from variations in the intrinsic conductivity of the material or could instead be attributed to a photoelectrochemical phenomenon. A custom device (Figure S5) was designed to position a copper contact on the film surface, with a second contact at the same location serving as the electrical connection during photoelectrochemical experiments. This configuration enabled electron transport from the FTO substrate to the film surface in a manner analogous to the electrochemical setup. For control and comparison, bare FTO electrodes and BiVO_4_ photoelectrodes were also measured under identical conditions.

Figure 11 shows the Bode plots under dark and illuminated conditions. Phase values close to zero, along with a nearly constant total impedance module, indicate a predominantly resistive behavior of the materials. At high frequencies, BiVO_4_ exhibited slight phase variations, although not exceeding 5°. The mean value of the total impedance module was calculated to evaluate the influence of illumination (Table S1). FTO displayed a 1.2% variation, BiVO_4_ a 3.1% variation, and BiO_*x*_ a 9.4% change, demonstrating that illumination had a significantly more substantial impact on BiO_*x*_ impedance compared to other materials.

**Figure 11:**
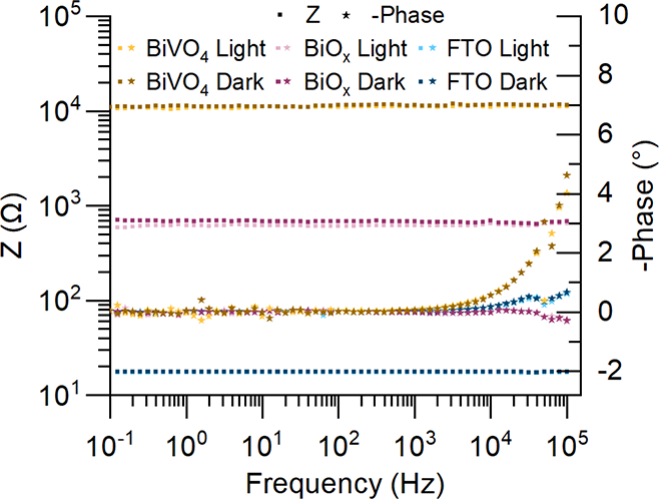
Bode plots from electrolyte-free impedance in dark and illuminated conditions. Impedance module (square) and phase (star) values. Parameters: *E*_DC_ = 0 V; amplitude = 100 mV (RMS); frequency range = 100 kHz to 0.1 Hz.

The result without an electrochemical inrterface suggests that the conductivity of BiO_*x*_ may increase under illumination; however, the variations observed in the electrolyte-free configuration were minor compared to those obtained from electrochemical impedance measurements. In the presence of an electrochemical interface, oxygen vacancies, ordinarily present in this class of materials, can reduce the overpotential required for OER by introducing excess electrons that influence adsorbate binding via surface-to-adsorbate charge transfer.^45^ This phenomenon is well documented in the literature for both electrocatalytic^46^ and photoelectrochemical^47^ OER. Our findings indicate that changes in conductivity alone cannot fully explain for the photoswitchable behavior of BiO_*x*_, and photoswitchable oxygen vacancies, similar to those reported for BiOCl,^29,30^ likely contribute synergistically to visible-light enhanced electrochemical water splitting performance.

## 3 Conclusions

The present results provide advances in BiO_*x*_ electrodeposition and report the unexplored photoswitchable properties of this material. Deposition time was reduced from 90 to 10 min using a xenon illuminator, while maintaining a similar amperometric profile. The use of a stainless-steel cathode further lowered material costs, supporting the feasibility of system scale-up. Anodic polarization in neutral media successfully reversed the spontaneous passivation of BiO_*x*_ to Bi_2_O_3_. Chopped light voltammetry revealed a photoswitchable enhancement in the electrocatalytic activity of BiO_*x*_ for water-splitting in a neutral electrolyte. Electrochemical impedance spectroscopy indicated that this behavior is primarily associated with a decrease in depletion layer resistance, while electrolyte-free impedance measurements also suggested changes in the intrinsic conductivity of the material. Although BiO_*x*_ is not capable of sustaining photocurrent without the contribution of dark current (an essential condition for an efficient photoanode), our findings demonstrate that illumination exerts a significant influence that cannot be overlooked when Bi^5+^ containing bismuth oxides are employed as anodes for electrocatalysis.

## 4 Methods

### 4.1 Materials

All reagents were analytical grade and purchased from Sigma-Aldrich (Merck Group). Solutions were prepared by diluting the reagents in deionized water (Millipore Direct-Q 3 UV Water Purification System). Fluorine-doped Tin Oxide (FTO) glass substrates were also purchased from Sigma-Aldrich (Merck Group).

### 4.2 BiO_*x*_ Electrodeposition

BiO_*x*_ electrodes were electrodeposited onto fluorine-doped tin oxide (FTO) substrates using a modified version of the procedure reported by Wang *et al*.^43^ Prior to deposition, the FTO substrates were cleaned in an ultrasonic bath for 15 min using a 1:1 mixture of acetone and ethanol. Electrodeposition was performed in a custom-designed electrochemical cell using a PGSTAT302N Potentiostat/Galvanostat (Metrohm Autolab), operated via NOVA software. The electrodeposition solution consisted of 0.2 M BiNO_3_·5H_2_O in HNO_3_ 0.63 and 7 M acetic acid. A three-electrode system was employed: the working electrode was an FTO (2 cm^2^ submerged area); the counter electrode was a stainless steel plate (4 cm^2^ submerged area), and the reference electrode was a saturated calomel electrode (SCE, 3 M Hg_2_Cl_2_). Unless stated otherwise, the electrodeposition was conducted for 90 min under a constant potential of 2.57 V vs Hg_2_Cl_2_.

Electrodeposition studies were conducted under chopped and constant illumination conditions using a Xenon Illuminator (Luzchem) equipped with matching filters and calibrated to achieve AM 1.5 G (100 mW cm^–2^ in the 315–850 nm region), as specified in ASTM G173-03. The PGSTAT302N (Metrohm Autolab) acted as a Potentiostat/Galvanostat and shutter controller for those experiments.

### 4.3 BiVO_4_ Synthesis

BiVO_4_ electrodes were synthesized on FTO substrates following the method described by Wang *et al*.^43^ The synthesis involved three main steps: (i) electrodeposition of a bismuth oxide precursor film (BiO_*x*_), as described in section BiO_*x*_ electrodeposition, (ii) application of a vanadium-containing solution followed by solvent evaporation at 100 °C, and (iii) annealing of the film at 500 °C.

A 0.1 M NH_4_VO_3_ solution was used as the vanadium source. Following complete dissolution, 80 μL of the solution was carefully drop-cast onto the surface of the BiO_*x*_-modified electrode. The solvent was subsequently evaporated on a hot plate at 100 °C. Annealing was performed at 500 °C, with a heating rate of 10 °C/min, and the temperature was maintained for 2 h. After annealing, the system was allowed to cool naturally to room temperature overnight. To eliminate any residual V_2_O_5_, the resulting BiVO_4_ films were rinsed with a 1 M NaOH solution.

### 4.4 Photoelectrochemical Measurements

Electrochemical Impedance Spectroscopy (EIS) and Chopped Light Voltammetry were employed to characterize the photoelectrochemical performance of BiO_*x*_ electrodes. EIS measurements were conducted prior to LSV to prevent surface modifications resulting from high polarization potentials. All measurements were carried out in a 1 M Na_2_SO_4_ electrolyte solution using a three-electrode configuration. The working electrode consisted of an FTO substrate modified with either BiO_*x*_ film. A platinum spiral electrode (4.5 cm^2^ area) served as the counter electrode, while the reference electrode was a 3 M Hg_2_Cl_2_ (calomel). The electrochemical experiments were performed using a redox.me electrochemical cell model MM FC PEC 15 mL single-sided with 0.2 cm^2^ of WE exposed area. This single-sided photoelectrochemical cell with the photoelectrode pressed into the cell wall enabled back-side illumination through the FTO substrate, while the photoanode surface with BiO_*x*_ remained in direct contact with the electrolyte. The electrochemical cell was irradiated under either chopped or continuous illumination using a Xenon light source (Luzchem), as previously described. Electrochemical impedance spectroscopy (EIS) measurements were conducted under both illuminated and dark conditions using a VIONIC Potentiostat/Galvanostat (Metrohm Autolab) controlled by Intello software. The open circuit potential (OCP) was recorded until stabilization before initiating the impedance measurement. Scan parameters: DC potential (*E*_DC_) = OCP, frequency range = 100 kHz to 10 mHz, 10 freq p/decade, amplitude of 20 mV (RMS). EIS data were fitted using nonlinear regression based on Equivalent Circuit Analog models. The model was based on the work of Lopes *et al*.^48^ Constant-Phase elements were converted to effective capacitance using eq 11.

$$C_{\mathrm{eff}}(F)={Y_{0}}^{1/N}.{\left[\frac{1}{R_{S}}+\frac{1}{R_{P}}\right]}^{N-1/N}$$ 11

where *Y*_0_ is the admittance from CPE, *N* is *N* from CPE, *R*_S_ = *R*_S_ for *C*_DP_ and (*R*_S_ + *R*_DP_) for *C*_H_, and *R*_P_ is the *R*_DP_ for *C*_DP_ and *R*_H_ for *C*_H_.

Chopped light voltammetry was employed to evaluate the current response under illuminated and dark conditions within a single voltammetric sweep. For these studies, a PGSTAT302N (Metrohm Autolab) acted as a Potentiostat/Galvanostat and shutter controller operated via NOVA software (Metrohm Autolab). Before the voltammetric measurements, the open-circuit potential (OCP) was recorded until stabilization. The linear potential sweep was carried out under the following conditions: initial potential (*E*_i_) = 0 V vs OCP, with the sweep being terminated at 2.55 V vs RHE. A scan rate of 1 mV/s and a potential step of 1 mV were used, with an illumination frequency set at 0.1 Hz. All potential values were converted to RHE using Equation 12).

$$E_{\mathrm{RHE}}=E_{\mathrm{calomel}}+0.244+(0.059^{*}\mathrm{pH})$$ 12

### 4.5 Electrolyte Free Impedance Spectroscopy

Electrolyte-free impedance spectroscopy measurements were carried out using a custom two-contact support device (Figure S5). The support consisted of two copper cylinders, each 2.25 mm in diameter, mounted within an acrylic holder. The acrylic support was designed to position the photoelectrode so that the back side could be exposed to illumination, consistent with the electrochemical experiments. To establish electrical contact while avoiding film damage, each copper cylinder was spring-loaded and gently pressed against the electrode surface with the aid of a contact screw. During the measurements, the separation between the copper contacts was fixed at 1.4 cm. Impedance measurements were performed using a Metrohm Autolab Potentiostat/Galvanostat model VIONIC equipped with Intello software. The experimental parameters were: DC potential (*E*_DC_) = 0 V, frequency range = 100 kHz to 10 mHz with 10 frequencies per decade, and amplitude of 100 mV (RMS).

### 4.6 X-ray Diffraction

The X-ray diffraction (XRD) technique was used to characterize the composition of the synthesized materials based on their crystalline structures. XRD measurements were obtained using an X-ray diffractometer from Rigaku, model Miniflex (*V* = 15 kV, *I* = 30 mA), with a Cu Kα radiation source (λ = 1.5418 Å). The angular range, 2θ, was scanned from 5–90°, with a counting time of 0.05 s. The photoelectrodes were fixed in a 3D-printed holder with a window to expose the face of the deposited material. The diffractograms used as references were obtained from the Crystallography Open Database (COD) Web site.

### 4.7 Scanning Electron Microscopy with Energy-Dispersive Spectroscopy

Scanning electron microscopy (FIB/SEM) of BiO_*x*_ films was performed using a dual-beam platform from FEI Company model Nova Nanolab 600. The SEM was performed using an accelerating voltage of 10 keV and an electron beam current of 0.13 nA, at a working distance of 5.0 mm. BiO_*x*_ films deposited on the FTO substrate had an area of 1 cm^2^. Following deposition, the films were rinsed with deionized water and then fixed onto aluminum stubs using carbon tape.

## Supplementary Material



## Notes

Morphology and elemental characterization: SEM with EDS spectra of BiO_*x*_ films; electrolyte free impedance spectroscopy: images of the developed photoelectrode support and calculated mean values from the impedance module (PDF)
