# Supplementary material for: Photoswitchable Activity of Mixed Bismuth Oxide (BiO x ) for Water Splitting in Neutral Media
Source: ACS Omega. 2026 Mar 4;11(10):16098–107. doi: 10.1021/acsomega.5c10847 (PMC13000579; doi:10.1021/acsomega.5c10847)
Supplement: Supplementary file 1 [file ao5c10847_si_001.pdf]

Supporting Information

Photo-switchable Activity of Mixed Bismuth Oxide (BiOx) for Water Splitting in Neutral Media

André Guimarães de Oliveira[1*], Aparecida Cristina Mauro[1], Marcus Vinicius David[2], Ana Maria Rocco[1*]

[1] Conductive Materials and Energy Group, Chemical and Biochemical Engineering Processes, School of Chemistry, UFRJ, Universidade Federal do Rio de Janeiro, Centro de Tecnologia, Bloco E, Rio de Janeiro, CEP: 21941-909, RJ, Brazil

[2]Materials Metrology Division, National Institute of Metrology, Standardization and Industrial Quality, Inmetro/Dimat, Av. N. S. Graças 50, Xerém, CEP 25250-020, RJ, Brazil

* Corresponding author email: andregdo@eq.ufrj.br, amrocco@eq.ufrj.br

EDS Analysis of the BiO_x Film

The chemical elements present in BiO_x films were determined using SEM microscale energy-dispersive X-ray spectroscopy (EDS). The measurements were carried out on a dual-beam platform from FEI Company model Nova Nanolab 600 with a Si(Li) diode detector system coupled with Genesis Spectrum software. BiO_x EDS spectra were collected at a beam voltage of 20 keV and 0.13 nA electron current at a working distance of 4 mm. Scans were taken on an area of approximately 132 µm².

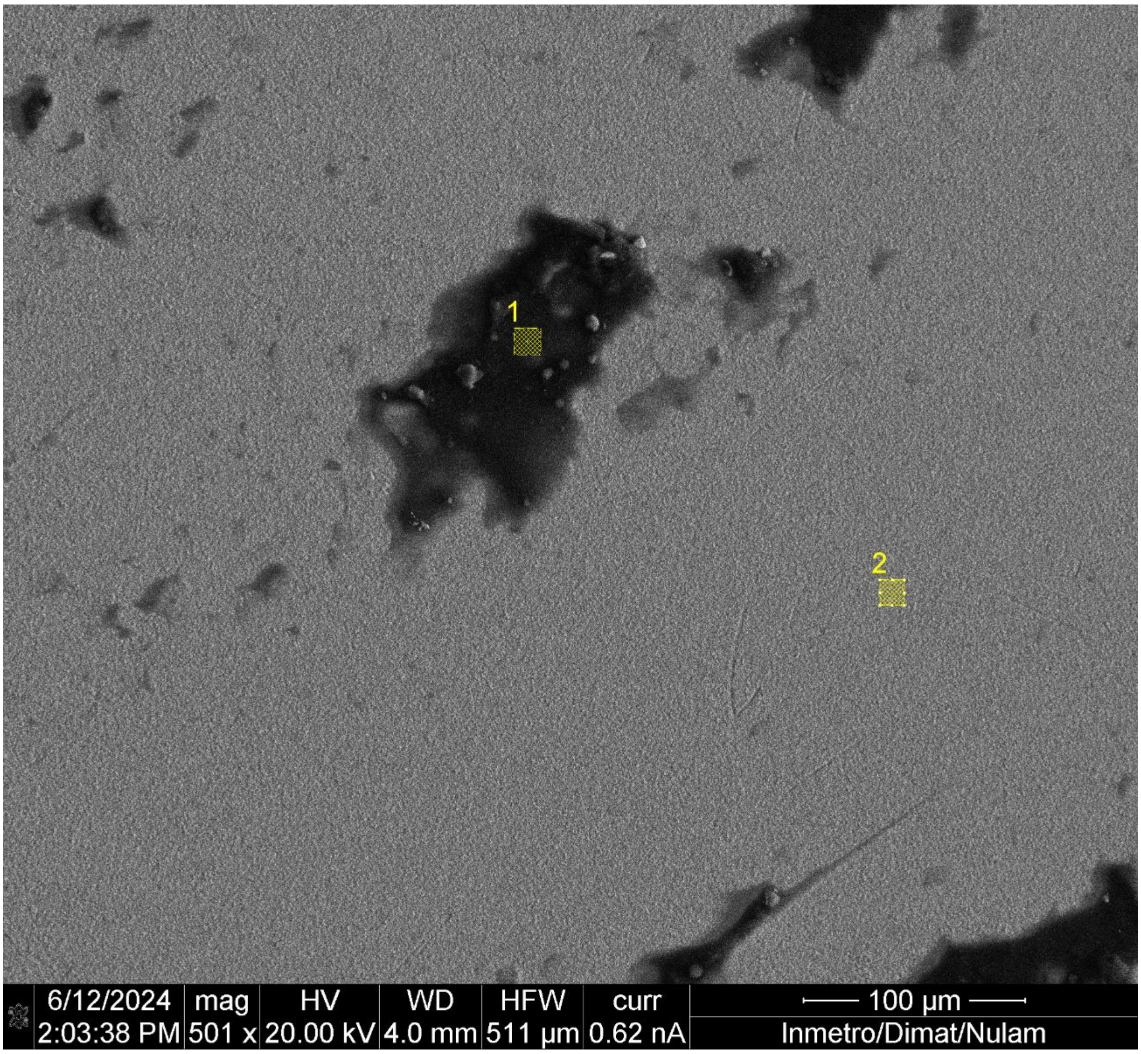

Figure S1. SEM image of BiO_x film at the region EDS spectra were obtained.

The following spectrum corresponds to the entire area displayed in the SEM image (Figure S1). It provides a general overview of the film, encompassing both the lighter regions, which represent the deposited material, and the darker spots.

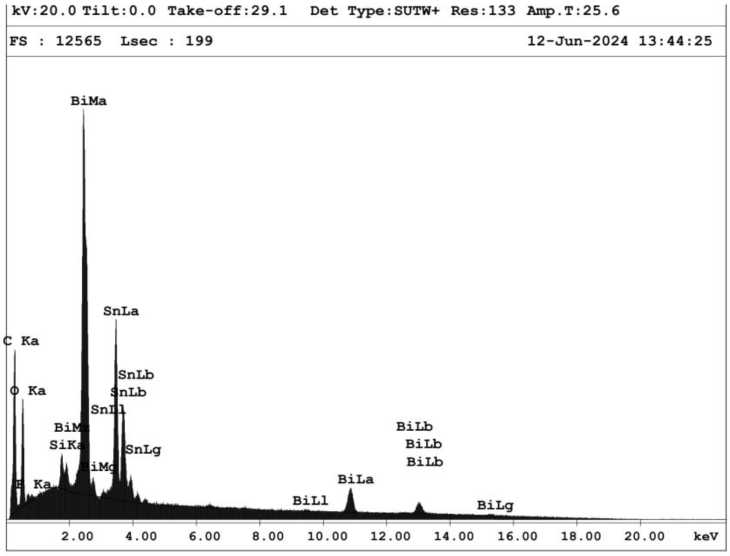

EDAX ZAF Quantification (Standardless)
Element Normalized
SEC Table : Default

Element	Wt %	At %	K-Ratio	Z	A	F
C K	23.86	61.96	0.0780	1.1805	0.2770	1.0001
O K	12.35	24.07	0.0212	1.1600	0.1480	1.0000
F K	0.94	1.54	0.0015	1.0898	0.1493	1.0000
SiK	0.89	0.99	0.0055	1.1084	0.5560	1.0012
SnL	19.28	5.07	0.1360	0.8719	0.8091	1.0000
BiL	42.68	6.37	0.3263	0.7520	1.0167	1.0000
Total	100.00	100.00				

Figure S2. EDS spectrum of the entire area displayed in Figure S1.

The image also highlights two small, numbered square areas (1 and 2), which were individually analyzed. Their respective spectra are shown below. The spectrum of region 1, corresponding to a dark spot, reveals more than 90 at.% carbon and less than 1 at. % bismuth. In region 2, the bismuth content increases significantly, while the carbon level decreases. These results support the hypothesis that certain areas were not covered during electrodeposition, likely due to surface contamination by carbon-rich materials.

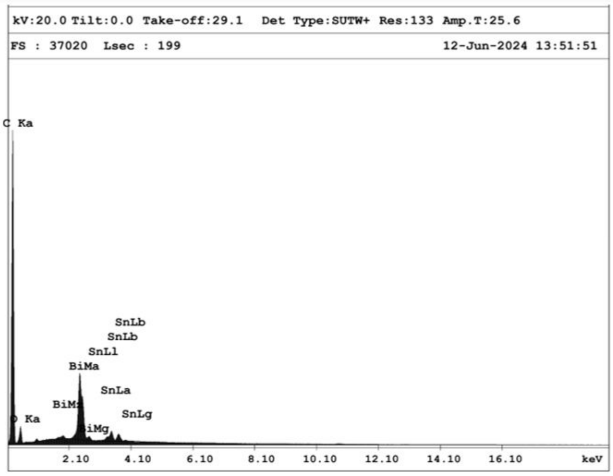

EDAX ZAF Quantification (Standardless)
Element Normalized
SEC Table : Default

Element	Wt %	At %	K-Ratio	Z	A	F
C K	76.37	92.50	0.4353	1.0363	0.5501	1.0000
O K	6.80	6.18	0.0095	1.0190	0.1377	1.0000
BiM	14.09	0.98	0.1344	0.6704	1.4225	1.0000
SnL	2.75	0.34	0.0221	0.7575	1.0635	1.0000
Total	100.00	100.00				

Figure S3. EDS spectrum of region 1 displayed in Figure S1.

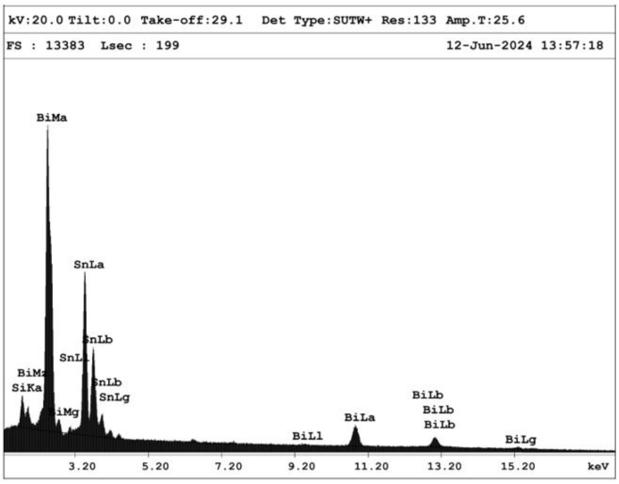

EDAX ZAF Quantification (Standardless)
Element Normalized
SEC Table : Default

Element	Wt %	At %	K-Ratio	Z	A	F
C K	10.49	39.74	0.0318	1.2413	0.2440	1.0001
O K	12.15	34.56	0.0215	1.2194	0.1449	1.0000
F K	1.32	3.15	0.0021	1.1454	0.1400	1.0001
SiK	1.24	2.00	0.0073	1.1636	0.5050	1.0014
SnL	25.65	9.84	0.1786	0.9195	0.7573	1.0000
BiL	49.16	10.71	0.4017	0.8077	1.0116	1.0000
Total	100.00	100.00				

Figure S4. EDS spectrum of region 2 is displayed in Figure S1.

Electrolyte-free Impedance Spectroscopy

Figure S2 displays 3D representations and a photograph of the device developed to support the photoelectrodes in two-point impedance spectroscopy.

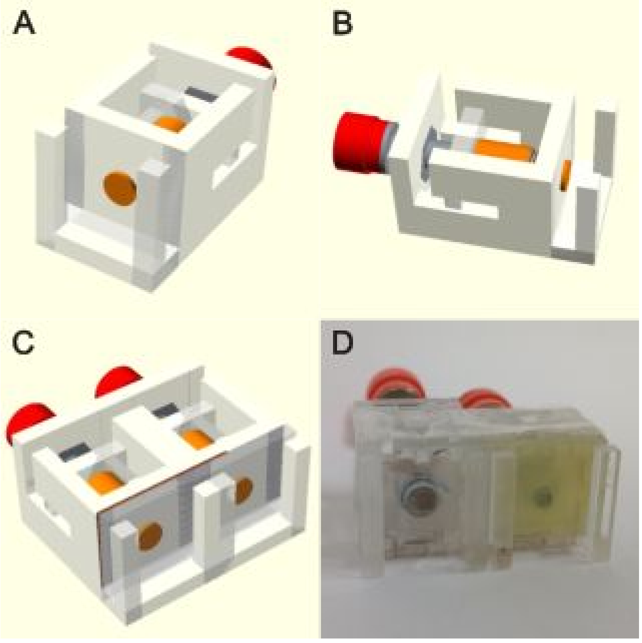

Figure S5. A and B: 3D representation of a single contact. C: 3D representation of the two contacts assembled with a photoelectrode. D: real photo of the device with a photoelectrode attached.

Table S1 presents the mean impedance module values obtained for BiO_x, $BiVO_4$, and FTO, along with their respective percentage variations, as determined by two-point impedance spectroscopy.

Table S1. Mean values of the total impedance module exhibited in Figure 11 along with the percentage variation between dark and illuminated conditions.

Electrode	Mean value Dark Z (Ω)	Mean value Light Z (Ω)	% Variation

FTO	17.53	17.32	1.2
$BiVO_4$	1.136E4	1.101E4	3.1
BiO_x	680.9	617.1	9.4
